# Acceptability, technological feasibility and educational value of remotely facilitated simulation based training: A scoping review

**DOI:** 10.1080/10872981.2021.1972506

**Published:** 2021-08-26

**Authors:** Robert Heffernan, Kay Brumpton, David Randles, Janani Pinidiyapathirage

**Affiliations:** a Rural Medical Education Australia, Toowoomba, QLDAustralia; b Griffith University, School of Medicine and Dentistry, Gold Coast, QLD, Australia

**Keywords:** Simulation-based training, remotely facilitated training, locally facilitated training, telesimulation, medical education

## Abstract

Although remote teaching and learning is not new to medical education, the Covid-19 pandemic has heightened its importance as a mode of education delivery. This scoping review aims to provide a narrative/iterative summary of the current literature in assessing the acceptability, educational value and technological feasibility of remotely facilitated (RF) simulation-based training (SBT) – ‘telesimulation’, for medical students and facilitators. The review was conducted using the method described by Arksey and O’Malley. A systematic process was followed to search multiple electronic databases supplemented with a general internet search to identify any relevant grey literature. The search strategy was developed in collaboration with medical students and educators familiar with SBT. Nine articles were identified as fitting the review inclusion criteria. The results indicated that RF SBT was positively viewed by participants but may not be viewed as equivalent to locally facilitated SBT. Participants of RF SBT felt confident to deal with common acute scenarios, believed it could expand their knowledge and skills and in turn would improve patient care in the clinical setting. Facilitators found RF SBT to be technologically feasible, promoting the acquisition of desired learning outcomes. Future research should assess the reaction to, and learning acquired during RF SBT, particularly, the perception and attitudes of facilitators. A clear research gap was identified in literature assessing the role of RF SBT in behavioural change and improved clinical care outcomes. Addresing these gaps will clarify the role of RF SBT in medical education.

## Introduction

The current COVID-19 pandemic has disrupted traditional medical educational delivery and is an opportunity to explore alternative models including remotely facilitated (RF) simulation-based training (SBT) – ‘telesimulation’. Suspension of face-to-face teaching activities such as practicals, laboratory sessions and cadaveric dissections has impacted training delivered to students [1]. The pandemic has thus highlighted the importance of establishing content which can be accessed by all learners. Online learning offers a solution to restricted population mobility alongside other benefits such as reduced costs, greater flexibility and self-paced learning [2,3]. The role and value of face-to-face simulation-based learning in medical education is well established with participants reporting increased self-assuredness to deliver improved quality of care [4]. SBT involves the immersion of participants in real world, contextually meaningful, artificially constructed scenarios using standardised patients or mannequins with pre- and post-simulation briefings [4,5]. The method uses a constructivist learning approach to teach higher order learning outcomes [4] and provides an opportunity to teach and practice in a safe environment without risks to patients [6,7]. RF SBT may therefore be appropriate for training in experiential scenarios such as crises, emergencies, and when in geographical isolation.

Telesimulation is the process by which telecommunication and simulation resources are used to deliver education and training to remote locations [7]. Remote or distance-based education helps to address the uneven distribution of medical education resources that can significantly impact on medical students’ access to education in rural areas [5,8]. Distance-based education has been delivered on several platforms such as iPhone® with FaceTime®, or an Android® based phone with Skype®, Yahoo Messenger® or Microsoft Messenger® [9]. There is published data covering the diversity of content, designs and technology used in distance-based education. Other teaching and learning modes such as adaptive and machine learning, augmented and virtual reality, and virtual simulations (e.g., Second Life) are also being investigated for remote learning [10–13], however, SBT differs from these other modes of training by undertaking observation of the candidate actively participating in scenarios and immediately reflecting on performance through debriefing [5].

Given the prevalence of social distancing protocols and regularity of lockdowns across the globe, there is a present need for a general assessment of the validity of telesimulation as an educational practice. Thus, we have undertaken a rapid review of the literature on telesimulation, defined here as remote facilitation or delivery of simulation-based training. The aim of this review is to assess some aspects of the validity of remotely facilitated simulation-based training as an educational practice by answering the research questions:

1. What is the acceptability and educational value of telesimulation for medical students?

2. What is the acceptability and technological feasibility of telesimulation for facilitators?

## Methods

The methods we used are similar to Arksey and O’Malley’s five-step process for conducting a scoping review [14]: 1) identify the research question, 2) identify relevant studies, 3) select studies, 4) chart data, and 5) collate, summarise and report.

### Search strategy and identification of relevant studies

A systematic search of electronic databases was performed in May 2020 using; MEDLINE, EMBASE, CINAHL, PUBMED, Scopus, MEDLINE OVID, Science Citation Index, Proquest Central, Web of Science, Google Scholar, and Springerlink Fully Open Access Journals. Databases were searched for literature published since 2009 in the English language using the following search terms in the Title/Abstract field:

- (‘medical educator’ OR ‘health professional educator’ OR ‘clinical educator’ OR teacher) AND
- (remote OR distant OR online) AND
- (simulation OR telesimulation OR ‘experiential learning’) AND
- (‘medical student’ OR student)

Note, clinical educator or teacher is defined here as professional experts in the development, delivery, and facilitation of education to undergraduate and postgraduate health students. A simple internet search was also performed using similar keywords to identify any relevant grey literature published during the same time period. Additionally, References of identified articles were manually searched to make sure that all relevant articles were included in the review. Studies addressing only the delivery of educational content over teleconference, or the monitoring and assessing of performance of shared activities over teleconference were excluded as they did not allow for visual demonstrations or assessment of student learning [1]. The search strategy was developed in collaboration with medical educators and medical students familiar with simulation, thus incorporating stakeholder involvement into the search strategy.

Results were presented using the Preferred Reporting Items for Systematic Reviews and Meta-Analyses (PRISMA) Extension for Scoping Reviews (PRISMA-ScR) [15].

### Data charting and abstraction

Each full text article was reviewed by a medical educator and medical student and the relevant key features were recorded. Kirkpatrick’s educational model was chosen as the structure for data extraction due to authors’ prior experience with the model [16]. The model and research questions were used to categorise the extracted data into the levels given below:

Level 1 (Reaction)

- Technical feasibility of telesimulation
- Student perception of telesimulation
- Facilitator perception of telesimulation

Level 2 (Learning)

- Telesimulation and teamwork
- Student changes in attitude
- Effectiveness of teaching learning outcomes using telesimulation

Level 3 (Behaviour)

- Behavioral changes following telesimulation

Level 4 (Outcomes)

- Changes in clinical outcomes following telesimulation

## Results

**Figure 1.**
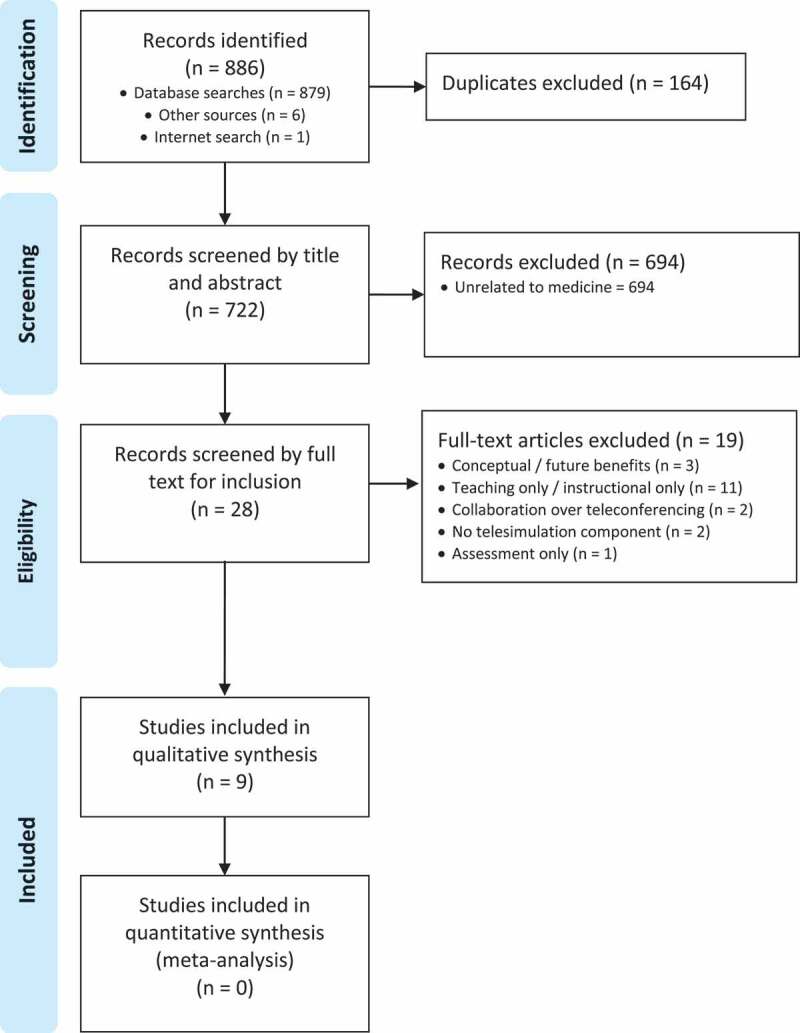
PRISMA Flowchart

The results of the systematic search are summarised in Figure 1. Nine articles were included in the final analysis from a total of 886 identified records (879 records were identified through database searches, one through a simple internet search and six through searching the reference lists of identified articles). After duplicates were removed, 722 records remained. These were screened by a medical educator and medical student to assess their relevance to the scoping review. After screening 28 records were assessed for eligibility, 19 of these records were then excluded as per the exclusion criteria.

All collected data related to level 1 (reaction) or level 2 (learning) of Kirkpatrick’s education model [16]. No identified studies related to level 3 (behaviour) or level 4 (outcomes). Key features of the included studies and their main findings are given in Table 1.

**Table 1.** Key features identified in studies using ‘telesimulation’ techniques

Author	Educational activity	Participants and assessment methods	Findings	
			Level 1 Technical feasibility and student/facilitator perception	Level 2 Teamwork, student changes in attitude, effectiveness of teaching learning outcomes
Ahmed et al. 2016 [20]	Simulated emergency training sessions for medicine residents that included teledebriefing or on-site debriefing	44 debriefing sessions using DASH 246 assessments	Teledebriefing was perceived as effective but the effectiveness was rated as lower than on-site debriefing (*p* < 0.001)	
Bolle 2009 [23]	Linked videoconferencing pairing 3 rural hospital emergency teams with 3 university hospital trauma teams	9 participants Semi-structured group interviews		Videoconferencing was found useful during emergencies and for developing virtual emergency teams
Christensen et al. 2015 [5]	Simulation scenarios delivered locally and remotely by videoconference to recently graduated doctors and nurses	305 Participants 16 questions (5-point Likert scale) measuring LF vs. RF experience Pre-post-test scores on knowledge	LF training demonstrated higher scores for participant learning experience compared to RF training (*p* = 0.01).	LF had a higher impact on teamwork and effectiveness of learning outcomes (*p* ≤ 0.04). Differences in pre- and post-test knowledge were similar for RF vs. LF (*p* = 0.65)
Collins et al. 2019 [17]	Traditional LF versus RF cardiopulmonary resuscitation training using videoconferencing for health students at two geographically separate sites	10 Participants 9 survey questions (5-point Likert scale)	RF environment was rated high for visual connectivity, facilitator audio connectivity and recommended for locations with low resource and facilitator availability	RF SBT met interprofessional learning outcomes, was an adequate replacement for traditional facilitation and a remote facilitator could provide a valuable simulation training experience using distance education
Hayden, Navedo and Gordon 2012 [18]	Web-conference software was used to deliver either standard in-person instructions or instructions delivered by a remote instructor to preclinical students caring for patients in a stimulation laboratory	44 surveys from 23 education events Seven questions (5-point Likert scale)	Communication was identified as a barrier to understanding the case delivered by the remote instructor (*p* < 0.0001). Willingness to participate in future RF sessions was lower compared to LF SBT (*p* = 0.0003)	No differences were observed between LF and RF SBT for the acquiring of new skills (*p* = 0.39) or knowledge (*p* = 0.41).
Ikeyama, Shimizu & Ohta 2012 [6]	Video recordings of stimulation scenarios (ventricular fibrillation or desaturation in an intubated patient) performed by healthcare providers at a training centre were transferred to a remote facilitator through the system for debriefing and interactive communication.	16 participants and 3 facilitators 2 pilot RF SBT sessions 5 online survey questions (5-point Likert scale)	Facilitators reported concerns they could not assess participants emotions and expression but gave a 5/5 rating of the ‘Operability’ of RF system.	Learning quality of RF was rated more effective than LF (94% vs. 87%) and 88% of the participants opted for RF training again.
McCoy et al. 2017 [21]	A live TV internet connection was used for communication and debriefing of medical students who remotely observed a live, fully immersive simulation case and its debriefing done by an on-site simulation group.	32 respondents LF and RF compared using a written evaluation tool	Evaluation scores of RF vs. LF were not different; RF 96.8% (95% Confidence Interval [CI] 94.8% – 98.9%) vs. LF 96.6% (95% CI 94.5% – 98.6%).	Learners reported that RF was an effective learning tool, beneficial to their education and enhanced patient care in clinical settings.
Ohta et al. 2017 [22]	Bidirectional live video communication between medical students allocated either to remote or on-site facilitation for paediatric resuscitation training.	136 participants in two groups Comparison made using a facilitator rated behavioral assessment tool (BAT) 7-survey questions (7-point Likert scale)	RF training was rated as a more effective facilitation format than LF (*p* = 0.004) and as more realistic (*p* = 0.049)	The observed improvement in BAT scores were similar between the two groups (*p* = 0.94).
Shao et al. 2018 [19]	Web conference software with recording capabilities was used to deliver remote, structured simulation training for clinicians from intensive care units in 7 countries.	18 participants 16 Sessions Performance assessed using a validated tool Satisfaction assessed with a survey (5-point Likert scale)	94% felt they were well prepared to manage common acute presentations following the training, 75% felt the training was realistic and 81% recommended training should be undertaken by all critical care team members.	Critical task completion rate significantly improved pre-post training (*p* = 0.002). Improvements post-RF training were also noted in code status, disability and review of home medication and allergies (*p* ≤ 0.01).

Level 1

### Technical feasibility of telesimulation

Five studies assessed the technical feasibility of delivering RF SBT [5,6,17–19]. Using a five-point Likert scale Christensen et al. [5] assessed participant reaction to RF SBT when compared with locally facilitated (LF) SBT. Questions related to feasibility of SBT found that participants rated the LF experience more positively than the RF experience (n _LF_ = 155, n _RF_ = 155, *p* < 0.05) [5]. Ikeyama, Shimizu and Ohta [6] using a five-point Likert Scale reported that the participants rated the quality of the RF sound system and the facilitator’s audibility, equivalent or better than that provided during LF SBT [6]. Despite these positive results, the study experienced interruption during the RF SBT due to unintended activation of ‘sleep mode’ on the computer, microphone requiring repositioning due to poor sound quality and limited viewing due to small screen size [6]. Collins et al. [17] conducted a pilot study using a five-point Likert scale and determined that set up and operation of telesimulation technology was sufficient for making good visual connectivity with the person speaking (n = 10, Mean *(M) *= 4.5 ± 0.7) and the facilitator audio connectivity was rated as very clear (n = 10, *M* = 3.9 ± 0.6). Hayden, Navedo and Gordon [18] also used a five-point Likert scale to compare RF and LF SBT and found communication between faculty and student was a barrier to understanding the case (n _RF_ = 25, RF = 2.8, 95% Confidence interval (CI) 2.4–3.2 vs. n _LF_ = 19, LF = 4.5, 95% CI 4.0 − 5.0; *p* < 0.001). Shao et al. [19] concluded that remote simulation is low cost, however there was no data to reflect this conclusion.

### Student perception of telesimulation

Positive participant experience was demonstrated in eight of the studies analysed [5,6,17–22], however, when comparing the perception of RF SBT to LF SBT, there were mixed results. Six studies demonstrated RF SBT to be equally favourable and effective when compared with LF SBT [6,19,21,22]. Ikeyama, Shimizu and Ohta [6] using a five point Likert scale found that 93.8% (15/16) of the participants rated RF SBT to be effective, 87.5% (14/16) rated RF SBT as equally or more effective than LF SBT whilst another 87.5% (14/16) indicated that they would like to receive RF simulation training again. McCoy et al. [21] used a written evaluation tool to compare the perception of LF and RF SBT and found no significant difference in mean evaluation scores between the two training modalities (n _LF_ = 32, LF 96.6%, 95% CI 94.5–98.6% vs. n _RF_ = 32, RF 96.8%, 95% CI 94.8–98.9%) [21]. The participants reported no difference in favourability between the two modalities and found RF SBT to be an equally effective learning tool beneficial to their education [21]. Ohta et al. [22] used a seven-point Likert Scale to compare RF and LF SBT. They demonstrated that Japanese medical students rated RF SBT format as more effective than LF SBT but emphasised that the difference was small (n _RF_ = 89, Median = 6, Interquartile Range (IQR) 6–7 vs. n _LF_ = 47, Median = 6, IQR 6–7; *p* = 0.004). It is worth noting that Ohta et al. [22] investigated effectiveness as a secondary outcome. The authors also reported that participants perceived simulation sessions by remote facilitation as more realistic than locally facilitated training (n _RF_ = 89, RF Median = 6, IQR 5–6 vs. n _LF_ = 47, LF Median 5, IQR 5–6; *p* = 0.049) [22]. Shao et al. [19] also used a five-point Likert scale to demonstrate that 75% of participants (n = 18) felt the training was realistic and 81% recommended training should be undertaken by all critical care team members. In a pilot study done by Collins et al. [17] using a five-point Likert scale, students (n = 10) reported that remote facilitation is an adequate replacement for traditional facilitation (*M* = 4.2 ± 0.6), remote facilitation could provide a valuable simulation training experience using distance education (*M* = 4.9 ± 0.3) and students recommended using telesimulation for locations with low resource and facilitator availability (*M* = 4.7 ± 0.7).

Other studies have reported RF SBT to be inferior to LF SBT [5,18,20]. Christensen et al. [5] used a 16 question five-point Likert scale to measure perceived learning, comfort, interaction with learners/instructors and quality of instruction of SBT. The authors found that LF SBT resulted in a significantly higher median total score when compared with RF SBT (n _LF_ = 155, LF Median = 78, IQR 72–80 vs. n _RF_ = 155, RF Median = 76, IQR 68–80; *p* = 0.01) [5]. Participants were also less positive about remote training when considering comfort, group interaction and instructor’s inquiry method [5]. Ahmed et al. [20] used the Debriefing Assessment for Simulation in Healthcare (DASH) – Student Version to compare participant perception of the effectiveness of debriefing in RF and LF SBT. Their results revealed a significant reduction in score for RF debriefing (n _RF_ = 30, RF *M =* 6.08 ± 0.57 vs. n _LF_ = 30, LF *M* = 6.64 ± 0.45; *p* < 0.001) [20]. Hayden, Navedo and Gordon [18] used a seven question five-point Likert scale to compare the impact of RF and LF SBT on the learner. They found that RF produced a significantly lower score when considering the following questions: was the communication between faculty and students a barrier to understanding the case? (n _RF_ = 25, RF 2.8, 95% CI 2.4–3.2 vs. n _LF_ = 19, LF 4.5, 95% CI 4.0–5.0; *p* < 0.0001) and would you participate again in such as session? (n _RF_ = 25, RF 4.2, 95% CI 4.6–5.2 vs. n _LF_ = 19, LF 4.9, 95% CI 4.6–5.2; *p* = 0.0003) [18].

### Facilitator perception of telesimulation

Only one study assessed facilitator perception of telesimulation [6]. Following RF SBT, Ikeyama, Shuizu and Ohta [6] collected verbal feedback from three facilitators for content analysis. The facilitators expressed concern about not being able to appropriately assess the emotions and facial expressions of participants [6]. The operability of the system was scored 5/5 by all three facilitators [6].

Level 2

### Telesimulation and teamwork

Bolle et al. [23] compared videoconferencing to teleconferencing and determined that videoconferencing was a useful method for developing emergency teams in the virtual environment based on semi-structured group interviews. Additionally, Collins et al.’s [17] pilot study used a five-point Likert scale to demonstrate that remote facilitation meets interprofessional learning outcomes (n = 10, *M* = 4.9 ± 0.3).

### Student changes in attitude

Three studies reported on changes in attitudes or perceptions of participants [18,19,21]. Shao et al. [19] used a five-point Likert scale to demonstrate that 94% (17/18) of clinicians from intensive care units felt well prepared to manage common acute presentations following a RF SBT program. McCoy et al. [21] evaluated the perceptions and attitudes of medical students towards their experience with RF and LF SBT and found that students believed both methods were equally beneficial in improving their ability to provide effective patient care in clinical settings . In addition, Hayden, Navedo and Gordon [18] found no difference between RF and LF SBT when assessing participants’ perception of acquiring new skills (n _LF_ = 19, n _RF_ = 25, *p* = 0.39) and knowledge (n _LF_ = 19, n _RF_ = 25, *p* = 0.41).

### Effectiveness of teaching learning outcomes using telesimulation

Three studies specifically assessed learning outcomes following RF SBT [5,19,22]. Ohta et al. [22] assessed team performance of simulation training participants measured by a behavioural assessment tool (BAT). Both remotely and locally facilitated groups improved their performance from the first to second scenario (n _RF_ = 89, RF first *M* = 8.5 ± 4.2; RF second *M* = 13.2 ± 6.2; *p* = 0.003 vs. n _LF_ = 47, LF first *M* = 6.9 ± 4.1, LF second *M* = 12.4 ± 6.4; *p* = 0.056) [22]. The difference in improvement between the two groups was not statistically significant (n _LF_ = 47, n _RF_ = 89; *p* = 0.94) indicating that the impact of remote facilitation on learning was similar to that of local facilitation [22]. The validated Checklist for Early Recognition and Treatment of Acute Illness (CERTAIN) [24] was used by Shao et al. [19] to assess the effect of 16 remote sessions of SBT. The authors found that critical task completion rate improved significantly (n = 18, 60.3% to 81.8%; *p* = 0.02) following completion of the SBT sessions. Specifically, improvement was shown in assessment of code status, disability, medical history, home medication and allergies [19]. Furthermore, a higher number of tasks were completed in the 10 minute post-simulation session and many tasks were completed much faster [19]. Christensen et al. [5] measured learning outcomes as a secondary measure by comparing RF SBT and LF SBT pre- and post-test scores and pass rates in a multiple-choice questionnaire. They found a significant difference in post-test scores between the two groups (n _RF_ = 154, RF Median = 13, IQR 12–14 vs. n _LF_ = 143, LF Median = 14, IQR 13–14; *p* < 0.05) [5].

Level 3 and 4

No identified studies related to level 3 (behaviour) or level 4 (results).

## Discussion

This is the first review to synthesise current evidence on the use of telesimulation. A systematic search strategy was used to identify nine articles which were classified according to Kirkpatrick’s educational model [16].

Four studies [5,6,16,18] assessed the technical feasibility of delivering remotely facilitated simulation-based training with mixed results. Ikeyama, Shimizu and Ohta [6] concluded that the technology was non-inferior, however they encountered a number of problems which could be easily overcome. The technology incorporated in the trials involving RF SBT are well established videoconferencing and smartphone platforms. Their reliability for use in communication is well established outside of SBT and it would be reasonable to assume this is transferable. Extensive investigation of these platforms in SBT has not yet been undertaken.

The results indicate that whilst student perception of RF SBT is largely positive, it is not always viewed as equal to LF SBT. Part of this difference could be explained by the environment created when using remote facilitation. Successful LF SBT requires facilitators to create a confidential environment where the participants feel safe, supported, valued and respected [25]. A similar environment must also be created for RF SBT [5]. Differences in the educational environment between locally and remotely facilitated SBT could be explained by the Social Presence Model [26]. The model suggests that social presence is essential for cultivating relationships and emotions that improve learning experiences [5,26]. Whilst an atmosphere to accommodate social presence can be created using LF SBT, it is unknown if this is possible in RF SBT. Christensen et al. (2015) propose that barriers for social presence could be overcome by improving or further developing facilitators’ interactive and communication skills. The optimal delivery method of RF SBT needs to be identified to ensure the creation of a psychologically safe and comfortable learning environments for participants.

Facilitators play a crucial role in the delivery of telesimulation. A key aspect of simulation is the use of high-quality facilitation followed by debriefing [6,20,25,27]. Debriefing involves a facilitated discussion with the promotion of learner self-reflection on performance [28]. Expert facilitators must understand the content being delivered, be knowledgeable in best practice techniques for debriefing and be able to establish a safe environment for learning and reflective self-discovery [20]. Debriefing is essential for participants to gain a more in depth understanding of learning outcomes [20,25]. Observation of the simulation provides the facilitator an opportunity to assess participant characteristics as well as team behaviours and interactions [25]. Three studies [5,18,20] found that remote interaction with the facilitator was perceived as inferior when compared with face-to-face interaction. It would be reasonable to assume that inferior remote facilitator interaction and debriefing would have a significant impact on the quality of a simulation-based educational activity. Therefore, clarifying the nature of participant perceptions of facilitator interactions is important, as local access to expert facilitators can be limited and telesimulation is a plausible method of overcoming this barrier [18,20,22].

Given the key role of facilitators, there is a paucity of research assessing the reaction of facilitators to telesimulation. Limited evidence [3] suggested that the operability/technical feasibility of telesimulation was rated as adequate, however further research into this should be undertaken to ensure the reproducibility of this result. Additionally, it remains unclear as to whether remote facilitation and debriefing is acceptable to facilitators.

Studies have demonstrated that face to face SBT is an effective and established tool in medical education for training in the critical elements of effective teamwork [27,29–32]. SBT is especially important in the teaching of high-risk infrequent clinical presentations in interprofessional education [33–35]. Only a few studies [17,23] have assessed remote telesimulation and teamwork. Whilst Bolle et al. [23] did not directly use an observation-feedback model, it incorporated the use of emergency-based simulation with remote facilitation. By demonstrating that virtual trauma teams can improve clinical work processes and confidence in participants, Bolle et al. have established that there is an implied benefit of delivering simulation training via telesimulation [23].

Improving medical students’ attitudes is a central component of andragogy [36] and delivering learning outcomes [16]. Three studies [18,19,21] demonstrated a positive change in attitude in participants’ undertaking RF SBT in the areas of preparedness [19], improving patient care [21] and acquiring new skills and knowledge [18]. Whilst this may not guarantee achievement of these outcomes, changes in attitude reinforces the value of RF SBT. The development of improved attitudes is an important step in improving behaviours and performance [16,36].

Existing research into the effectiveness of RF SBT have been positive, with results indicating that students can have similar or improved educational outcomes and experiences through RF compared to LF SBT. Use of validated tools to demonstrate an improvement in performance is [19] encouraging and this was supported by two other studies [5,22]. The overall goal of medical education is to ensure students acquire the knowledge, skills and behaviours to prepare for clinical practice [37]. Studies have demonstrated an improvement in performance [5,19,22], and achievement of learning outcomes [5] however, a deeper understanding of the specific knowledge, skills and behaviours acquired is required.

Studies relating to level 3 and 4 of Kirkpatrick’s educational model were not found. Lack of data at these levels reflects the fact that telesimulation in medical education is still an emerging topic. While studies have shown that RF SBT shows promise as a medical education tool, further research is required to validate this method of instruction. Future research into RF SBT should: evaluate optimal learning environment design, investigate the role of RF SBT in developing participant’s teamwork skills, provide evidence of participants acquisition of knowledge and skills, explore participant perceptions of facilitator interactions and the acceptability of this delivery method to facilitators. Demonstration that RF SBT positively impacts on medical students’ behaviours and patient clinical outcomes will also be necessary to establish this pedagogical mode in medical education. Given the limited literature investigating RF SBT in medical education, future reviews would benefit by its applications in nursing and allied health professions.

The breadth of literature examining RF SBT is quite limited and varied in methodology. Consequently, this paper was not able to draw quantitative conclusions through metanalysis. The included studies also varied widely in terms of sample size and thus, their statistical power. Multiple studies relied on participant and facilitator feedback based predominantly upon Likert scale ratings. Using rating scales to obtain student feedback could be impacted by bias due to interpersonal student teacher relationships. For instance, positive scores may be given for reasons such as politeness. As such we would strongly encourage future studies – particularly those with low sample sizes – to have controls in place to ensure that the data collected genuinely reflects participant/facilitator experiences. The variability in control and intervention group construction was also of concern, for instance, in some studies groups were arranged by convenience or a subset of students participated in both control and intervention groups. Variability in group construction can reduce the reproducibility of studies while also limiting the chances of their inclusion in future metanalyses. Future research of RF SBT should aim to include sufficient sample sizes evidenced by statistical power calculations, use robust and reproducible methods to construct control and intervention groups and provide evidence of controls of bias when reporting Likert scores of patient/facilitator experiences.

## Conclusion

The aim of this scoping review was to explore the acceptability and educational value of remotely facilitated simulation-based training to students and its acceptability and technological feasibility for facilitators. Our results suggest that participants view RF SBT as a positive learning experience, though it may not be viewed as favourably as LF SBT. Following RF SBT participants reported increased confident in dealing with common acute scenarios, felt they would deliver improved patient care in clinical settings and believed they improved their knowledge and skills. Facilitators found RF SBT to be technologically feasible, promote the acquisition of desired learning outcomes, effective for interprofessional development and potentially to be useful when building virtual teams. Limited evidence was available to suggest that RF SBT is acceptable to facilitators.

RF SBT shows promise as a potentially valuable method of remote learning. However, further research is required to explore the exact role and benefits that RF SBT could contribute to medical education. In particular, it must explore the perception and attitudes of facilitators. Until research in these areas have been completed, the role of RF SBT in distance education will be unclear. Of highest importance is the need for research into higher level outcomes [16] to ensure RF SBT results in behavioural change and improved clinical care.

*Abbreviations: Remotely Facilitated (RF); Locally Facilitated (LF); Simulation-Based Training (SBT); DASH – Debriefing Assessment for Simulation in Healthcare*
